# Supplementary material for: Diagnostic Accuracy of CareStart™ Malaria HRP2 and SD Bioline Pf/PAN for Malaria in Febrile Outpatients in Varying Malaria Transmission Settings in Cameroon
Source: Diagnostics (Basel). 2021 Aug 27;11(9):1556. doi: 10.3390/diagnostics11091556 (PMC8469216; doi:10.3390/diagnostics11091556)
Supplement: Supplementary file 1 [file diagnostics-11-01556-s001.zip › diagnostics-1289318-supplementary.pdf]

Supplementary Material Manuscript Ali IM et al,

Table S1 Comparison of mRDT tests performances between StVC Dschang and DH Dschang (GS: Microscopy)

Index tests	Parameters	StVC Dschang	DH Dschang	Pearson's chi^2	*p*
Pf RDT	Sensitivity	0.65 (0.51 0.77)	0.17 (0.10-0.26)	35.53	< 0.001
	Specificity	0.91 (0.86, 0.95)	0.77 (0.64-0.87)	6.93	0.008
Pf +PAN RDT	Sensitivity	0.56 (0.43-0.68)	0.23 (0.17-0.31)	14.81	< 0.001
	Specificity	0.92 (0.86-0.96)	0.75 (0.65-0.83)	9.32	0.002

GS: Reference test.

Table S2: Comparison of pfRDT performances between CBCHS-IHC Ngounso and DH Dschang

GS	Parameters	CBCHS-IHC Ngounso	DH Dschang	Pearson's chi^2	*p*
Microscopy	Sensitivity	0.92 (0.86-0.96)	0.14 (0.08-0.23)	128.34	< 0.001
	Specificity	0.94 (0.89-0.97)	0.71 (0.57-0.83)	12.56	< 0.001

GS: Reference test.

Table S3: Comparison of pfRDT performances between Yaoundé and StVC Dschang

GS	Parameters	Yaoundé	StVC Dschang	Pearson's chi^2	*p*
Microscopy	Sensitivity	0.94 (0.91-0.97)	0.65 (0.51 – 0.77)	41.40	< 0.001
	Specificity	0.91 (0.85-0.96)	0.91 (0.86-0.95)	0.003	0.9
PCR	Sensitivity	0.91 (0.87-0.94)	0.56 (0.43-0.68)	49.82	< 0.001
	Specificity	0.97 (0.92-0.99)	0.92 (0.86-0.96)	3.36	0.06

GS: Reference test.

Table S4: Comparison of pf mRDT performances between Yaoundé and DH Dschang

GS	Parameters	Yaoundé	DH Dschang	Pearson's chi^2	*p*
Microscopy	Sensitivity	0.94 (0.91-0.97)	0.14 (0.08-0.23)	208.39	< 0.001
	Specificity	0.91 (0.85-0.96)	0.71 (0.57-0.83)	7.70	0.005

GS: Reference test.

Information sheet and Consent form

V 1.0

MOLECULAR SPECIATION OF MALARIA PARASITES IN FEBRILE PATIENTS CONSULTING AT HEALTH FACILITIES IN CENTRE AND WEST REGION OF CAMEROON

BACKGROUND

Your child has been sick with a fever and the doctor suspects it is due to malaria. Malaria can be caused by five types of parasites, which are very tiny and live inside red blood cells. Only three of these parasites are known to cause malaria in Cameroon, but there is some evidence that a fourth one, known as *Plasmodium vivax*, can be causing malaria also in Cameroon. In the Dschang district hospital, some researchers checked the blood of sick people and found that this some of the malaria patients had *Plasmodium vivax* malaria. But a big majority of the patients who had fever did not have malaria. The doctors should know what is causing their fevers. Although the current treatment in this hospital works for *P. vivax* like for other malaria parasites, it is important to know who are having these kind of parasites, where they come from and what they are exposed to before they get this parasite. In this study, we will work to understand using several methods if this parasite is still infecting people and to know what other infections cause fevers among people who come to consult here. The following paragraphs will explain better what will happen during the study and what happens to you when you chose to participate in this study which is carried out by researchers from the University of Dschang the national malaria control progam in Yaoundé.

BEING IN THE STUDY IS YOUR CHOICE

This information sheet tells you about the study. This information will be discussed with you. Once you know about the study and decide if you want your child to be in this study, you will be asked to sign this consent form and we will give you a copy to keep.

Before we tell you more about the study, you should know that:

- It is your choice to allow your child to be in the study.
- If you choose to allow your child to be in the study, you can withdraw him/her at any time.
- If you choose to allow your child to be in the study, you don’t have to answer any questions that you don’t want to.
- You may choose not to allow your child to join the study.
- If you choose not to allow your child to be in the study, your child will still get his/her regular tests and the usual care from the hospital workers.

<u>The goal of this evaluation/study is to:</u>

Primary goal:

To determine the prevalence of *Plasmodium spp* of circulating in Western highlands using molecular and point of care diagnostics in the Centre and West regions of Cameroon. .

Secondary goals

1. To compare RDT types against microscopy and PCR for the confirmation of malaria infections in the Yaounde, Dschang and Ngounso health district in the centre and West regions of Cameroon.
2. Characterize the genetic diversity of circulating malaria parasites
3. Use serologic analysis to identify people exposed to malaria and other causes of febrile illness.

If you choose to be in the study, we will collect blood from the tip of your child's finger just once.

WHAT HAPPENS DURING THE STUDY

- When your child joins the study, a member of the study staff will ask you questions about your child's background, age, illness and medication history and where you have been in the past three months. Participation in the study is once.
- For the study, about 4 drops (0.5ml) of blood will be taken from a finger in your child's hand when he/she joins the study.
- As part of regular care children with fevers are regularly prescribed blood tests for malaria. If your child's doctor requests these to be done, the blood will be taken at the same time as the blood for the study test which will be used for processing.
- . Being in this study will take a little extra time but just once as we will see once only.
- All the information will be kept private.
- All specimens in the study will not contain names of people taking part in the study. Instead, we will use a code that nobody out of the study will know.

IF YOU CHOOSE TO TAKE YOUR CHILD FROM THE STUDY

You can choose to take your child from the study at any time. This will not affect his/her health care he/she will get in the future in this hospital/clinic.

RISKS TO YOUR CHILD

There are minimal medical risks to your child other than a possible mild discomfort or bruising as can be expected with capillary (finger stick) blood collection procedures. Additionally, there is no risk of disclosure of confidential personal health information because the specimens will be de-linked upon enrollment in the study.

BENEFITS TO YOUR CHILD

There are no direct benefits to your child for participation in this study other than the opportunity to contribute to important knowledge that will be used to make the fight against malaria more successful. We will give you results of your test by microscopy or RDT. Other results will be made available to the hospital/university, malaria program once they are available.

REASONS FOR TAKING YOUR CHILD OUT OF THE STUDY WITHOUT YOUR CONSENT

Your child may be taken out of the study without your consent if:

- Your doctor decides that being in the study may cause harm to him/her;
- The study is stopped by the University, or the Ministry of Public Health of Cameroon.
- Other reasons beyond our control.

COSTS TO YOUR CHILD

- There is no cost to your child for participating in the study since the study visits are at the same times as his/her routine follow-up visits, we will not give transport to clinic visits.
- You will not be reimbursed for time or effort of being in this study.

YOUR CHILD'S RECORDS WILL BE PRIVATE

Your child's research records will be kept private as allowed by the law. Your child's study records will have a code number. Any information from your records will not be given out unless you agree in writing. Your name will not be in any reports about this study. People who review the study for us may need to look at your child's study records from time to time. For study needs, we will make copies of your medical records. All copies will be kept in a locked file cabinet. Only study staff will have access to this cabinet.

BLOOD SAMPLES

All your child's blood samples will be processed in the hospital laboratory for the duration of the study. Blood spotted on filter paper will be used only for the purpose of this study as described in the protocol. They will be destroyed after the testing according to the routine procedures.

PROBLEMS OR QUESTIONS

For questions about this study or a research-related injury, contact:

- Innocent Ali at the University of Dschang. phone number: 677021275 Email: dr.alinn@gmail.com
- Pr Kuiate Jules Roger, Head of Department, Biochemistry, University of Dschang. Email: jules.kuiate@univ-dschang.org ; phone number:
- For questions about your rights as a research subject, contact: IRB.

STATEMENT OF CONSENT:

Please sign your name below once you:

1. have read (have been explained) and know the reasons for the study,
2. know the steps to be followed in the study,
3. know the risks and benefits to your child for being in the study,
4. AND choose to enroll in this study of your own free will.

**Child's Name**<br>**Type or print**				

**Name of Child's Parent or Legal Guardian**		**Legal Guardian's Signature**		**Date**

**Witness's Name**<br>***Type or print***		**Witness's Signature**		**Date**

I have explained the purpose of this study to the volunteer. To the best of my knowledge, she understands the purpose, procedures, risks and benefits of this study.

**Investigator/Designee Name**<br>***Type or print***		**Investigator/Designee Signature**		**Date**

NOTE: This consent form with original signatures must be retained on file by the principal investigator. A copy must be given to the volunteer.

Supplemental Material

Figure S1

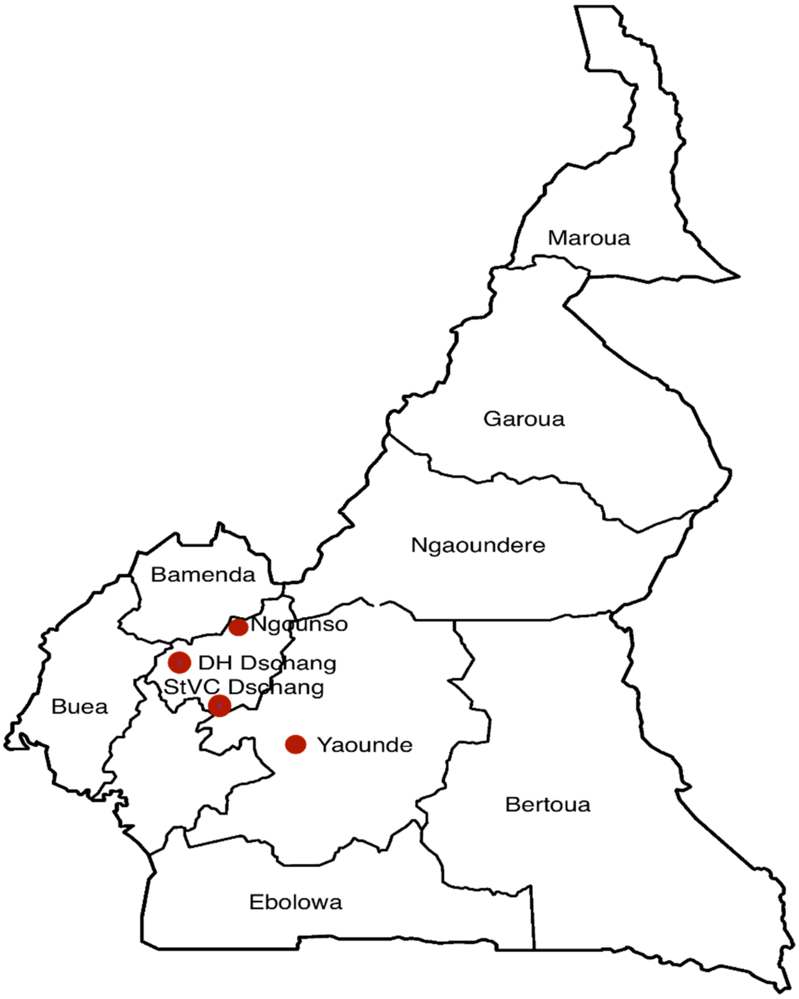

Figure S1: A map of the location of study sites.

Study sites are marked with a red dot while other map divisions represent boundaries of the ten regions of Cameroon, depicted by their capitals in each map division. Source of base map. The copyright holder of the blank map published file is licensed under the *Creative Commons* ***Attribution-Share Alike 3.0 Unported*** license.
